# Effects of antenatal nutrition counselling on dietary practices and nutritional status of pregnant women: A quasi-experimental hospital based study

**DOI:** 10.12669/pjms.36.4.1919

**Published:** 2020

**Authors:** Rameeza Kaleem, Muhammad Adnan, Mahnaz Nasir, Tayyaba Rahat

**Affiliations:** 1 Dr. Rameeza Kaleem, MBBS, MMCH, MD. HOD Social & Preventive Paediatrics, Department of Social & Preventive Paediatrics, Fatima Jinnah Medical University, Lahore, Pakistan; 2 Mr. Muhammad Adnan, M.Sc. Research Officer, Pakistan Health Research Council (PHRC) Research Center, Fatima Jinnah Medical University, Lahore, Pakistan; 3 Dr. Mahnaz Nasir, PhD. HOD Food Science and Human Nutrition, Department of Food Nutrition,Kinnaird College for Women, Lahore, Pakistan; 4 Ms. Tayyaba Rahat, M. Phil, Statistical Officer, Pakistan Health Research Council (PHRC) Research Center, Fatima Jinnah Medical University, Lahore, Pakistan

**Keywords:** Counselling, Dietary supplements, Pregnancy, Prenatal care, Vitamin D

## Abstract

**Objective:**

To assess the effects of nutrition education intervention on dietary practices and nutritional status of pregnant women.

**Methods:**

In this quasi-experimental study, 215 pregnant women were enrolled from Gynae OPD, Sir Ganga Ram Hospital, Lahore, Pakistan during 2017-18. Dietary practices were assessed using the usual intake form and scoring was done against food guide pyramid. Nutritional status was evaluated by anthropometric measurements and biochemical estimation. In addition to nutrition counselling, each woman was prescribed with supplements commonly used during pregnancy. Dietary habits and nutritional status were reassessed after two months. The women lost to follow up were 21 (9.8%) therefore data obtained from 194 women were subjected to final analysis by using SPSS 20.

**Results:**

The age of women ranged between 18 and 38 years. Those who never attended a school were 14.4%; poor 46.0%; and working 3.7%. The comparison between pre- and post-counselling dietary practices showed improvement in the numbers of women taking recommended portions of bread & cereals (79.4% vs. 95.9%, p = <0.001); vegetables (50.5% vs. 64.9%, p = 0.004); milk & dairy products (38.1% vs. 81.4%, p = <0.001); and a reduction in the numbers of women taking recommended portions of meat & bean (100.0% vs. 94.8%, p = 0.002). The frequency of women taking recommended diet as per food guide pyramid improved from 3.1% to 37.1%. Vitamin D status also showed improvement in the numbers of women with normal levels of serum vitamin D (7.1% vs. 33.3%, p = 0.079).

**Conclusions:**

Overall, nutrition counseling showed positive effects on nutritional status of pregnant women. Thus, the nutrition counseling must be an essential part of antenatal care for all pregnant women in the setting.

## INTRODUCTION

Pregnant women have higher nutritional requirement; whereas poor dietary practices in terms of food frequency and quality may cause nutritional deficiencies.^1^ Several studies have evaluated the association between prenatal dietary practices and pregnancy outcome.^2^-^4^ In maternal outcomes, it is associated with gestational weight and risk of anemia. In fetal outcomes, it is associated with birthweight and risk of preterm birth. Thus, healthy eating habits are essential for the wellbeing of pregnant mothers and better pregnancy outcome.^5^

Vitamin D deficiency (VDD) is a widespread micronutrient deficiency; and it may put pregnant mothers at risk of developing gestational diabetes mellitus,^6^ preeclampsia,^7^ and can increase the risk of preterm labor, poor fetal growth, and adverse neonatal outcome.^8^ National nutrition survey 2011 revealed that 51% pregnant Pakistani women were anaemic, 46% vitamin A deficient and 68.9% suffering from vitamin D deficiency.^9^

Nutrition counselling is a recommended approach to improve nutritional status of pregnant women. The WHO recommends that pregnant mothers should be supported to eat healthy and balanced diet for preventing nutritional deficiency.^5^ The reviews of literature show that several studies had evaluated the pregnant women’s knowledge, practices, and source of information regarding diet in pregnancy. Very few studies assessed the effects of nutrition counselling on their dietary practices and nutritional status. Therefore, this study was aimed to assess the effects of nutrition counselling on dietary practices and nutritional status of pregnant women seeking antenatal care at a public sector hospital.

## METHODS

The quasi-experimental study was carried out at Sir Ganga Ram Hospital, Lahore, Pakistan from September 2017 to February 2018. Ethical clearance No.09-Medicine/IERB dated 04.07.2017 was obtained from Institutional Ethics Review Board, Fatima Jinnah Medical University/ Sir Ganga Ram Hospital, Lahore, Pakistan. Informed written consents were taken from all volunteer pregnant women. Total 215 pregnant women of gestational age 19-29 weeks, visiting the facility for the first time to seek antenatal care, were enrolled by non-probability purposive sampling technique. Pregnant women reporting any illness or taking supplements before enrollment were excluded.

At baseline, all pregnant women were interviewed for demographic characteristics such as age, gestational age, education, family income, etc.; and subjected to the measurements of body weight, height, hemoglobin level, and vitamin D level etc. Routine antenatal checkup was done by a consultant gynaecologist and prenatal supplements were prescribed. Anthropometric measurements were done by trained lady health visitor. Mid upper arm circumference (MUAC) of the left upper arm at the midpoint between the tip of the shoulder and the tip of the elbow was measured by using MUAC tape. Serum vitamin D levels were estimated by using ELISA method. Normal vitamin D levels ranged between 30-100 ng/mL, insufficient between 20-30 ng/mL, and deficient < 20 ng/mL. A clinical nutritionist took detailed dietary history using usual intake form. According to their dietary assessment, nutritional counselling was done and diet charts purposefully designed for the study were provided.

All participants were followed up for two months. Then, a second interview was conducted in which post-counselling data were collected. Compliance with advised dietary practices and supplements was assessed. Women who were taking recommended servings from all five groups were labelled as having appropriate dietary practices. Similarly, a second blood specimen was collected to measure the improvement in vitamin D level.

Statistical Package for Social Sciences (SPSS) version 20 was used for data analysis. The women lost to follow up were 21 (9.8%) therefore data obtained from 194 (90.2%) women were subjected to final statistical analysis. Numerical variables such as age, MUAC and vitamin D levels were described by using mean ± standard deviation form. Categorical variables such as demographic characteristics and food frequency were discussed in frequency (percentage) form. Pre- and post-counselling dietary practices and vitamin D status were compared by using chi square test. P-value ≤0.05 was considered significant.

## RESULTS

The mean age of 194 pregnant women was 26.4±4.8 years (ranged between 18 and 38 years). The women who never attended a school were 14.4%; under-matric 27.5%; and graduate 18.1%. Only 3.7% women were doing a job. The assessment of family income per month showed that 46.0% women had earnings of PKR 20,000 or less; and others had between 20,000 and 50,000 PKR. The women experiencing first pregnancy were 3.3%. Overall, mean MUAC was 27.81±4.63 cm.

The comparison between pre- and post-counselling dietary practices showed improvement in the numbers of women taking recommended servings of five food groups as shown in Table-I. The frequency of women taking bread & cereals servings equivalent or above recommendation was further improved from 79.4% to 95.9% (p=<0.001). Likewise, one half of the total women were taking recommended servings of vegetables, and the numbers were significantly increased from 50.5% to 64.9% (p=0.004). Though, the frequency of women with appropriate intake of milk & dairy products was the lowest among the food groups under-investigation, but a remarkable post-counselling development was revealed (38.1% vs. 81.4%, p = <0.001). In meat and bean group, an unexpected and significant reduction in the numbers of normal practicing women was observed (100.0% vs. 94.8%, p = 0.002). A slight increase in the numbers of women having plenty of fluids could be achieved (74.2% vs. 78.4%, p = 0.339). Overall, the frequency of women taking recommended diet as per food guide pyramid was improved from 3.1% to 37.1%.

**Table-I:** Comparison of pre- and post-counselling dietary practices.

FOOD GROUPS		Pre-counselling (n=194)	Post-counselling (n=194)	p-value
Bread, cereals, pasta, rice and potato group (6-11 servings per day)	Recommended or above	154 (79.4%)	186 (95.9%)	<0.001
	Below recommendation	40 (20.6%)	08 (4.1%)	
Vegetables group (3-5 servings per day)	Recommended or above	98 (50.5%)	126 (64.9%)	0.004
	Below recommendation	96 (49.5%)	68 (35.1%)	
Milk and dairy products group (Aim to eat 3 servings per day)	Recommended or above	74 (38.1%)	158 (81.4%)	<0.001
	Below recommendation	120 (61.9%)	36 (18.6%)	
Fish, poultry, meat and bean group (Aim to eat 2 servings per day)	Recommended or above	194 (100.0%)	184 (94.8%)	0.002
	Below recommendation	00 (0.0%)	10 (5.2%)	
Fruits group (2-4 servings per day)	Recommended or above	80(41.2%)	130(67.0%)	<0.001
	Below recommendation	114 (58.8%)	64(33.0%)	

The assessment of supplement use showed that 19.6% women did not take any supplement. Among supplement users, 70.1% women used iron, 55.5% used calcium, 50.5% used folic acid, and 44.8% used vitamin D. When participants were categorized into two age groups, it was revealed that the age group 18-35 years had significantly lower mean levels of vitamin D than of age group 36-49 years (16.5±8.22 vs. 31.0±9.54); however, post-counselling mean vitamin D levels significantly improved from 16.5±8.22 to 23.82±15.25 (p = <0.001) within age group 18-35 years. Pre- and post-counselling mean levels of vitamin D were low in both literate and illiterate groups and the difference was not significant. Within group, both literate (14.4±2.5 vs. 27.3±15.5, p = 0.029) and illiterate (17.3±19.0 vs. 23.9±15.8, p = 0.021) revealed significant improvement. Pre- and post-counselling mean levels of vitamin D were also low in both poor and low-middle income groups and the difference was not significant. Within group, both poor (17.8±10.0 vs. 23.9±15.3, p = 0.027) and low-middle income group (16.0±6.8 vs. 24.8±16.6, p = 0.004) showed significant improvement.

Pre-counselling frequencies of vitamin D deficiency and insufficiency were 73.8% and 19.0%, respectively. None of the participant had vitamin D level above normal. Post-counselling frequencies of vitamin D deficiency and insufficiency were 35.7% and 31.0%, respectively. The comparison between pre- and post-counselling vitamin D status showed improvement in the numbers of women with normal levels of serum vitamin D (7.1% vs. 33.3%, p = 0.079). Table-II

**Table-II:** Comparison of pre- and post-counselling vitamin D status.

Vitamin D status	Pre-counselling (n=194)	Post-counselling (n=194)	p-value
Deficient (<20 ng/ml)	143 (73.8%)	69 (35.7%)	0.079
Insufficient (20-29 ng/ml)	37 (19.0%)	60 (31.0%)	
Normal (30-100 ng/ml)	14 (7.1%)	65 (33.3%)	
Toxicity (>100 ng/ml)	00 (0.0%)	00 (0.0%)	

## DISCUSSION

The World Health Organization (WHO) recommends the use of nutrition education and counselling to improve the nutritional status of pregnant women.^5^ Therefore, the present study evaluated the effects of nutrition counselling on dietary practices and nutritional status of pregnant women. Zelalem et al. assessed pregnancy specific dietary knowledge and practices of pregnant women of Addis Ababa and reported improvement in knowledge (53.9% vs. 97.0%) and practices (46.8% vs. 83.7%) after nutrition education intervention by healthcare providers.^10^ Similarly, Fallah et al. evaluated awareness level of Iranian pregnant women and observed significant improvement after nutrition education intervention (3% vs. 31%, P = <0.001). However, the progress in awareness level was independent of maternal age, education and obesity.^11^ Diddana et al. also provided nutrition education based on Health Belief Model to Ethiopian pregnant women and reported significant improvement in good dietary practice (56.5% vs. 84.1%, P = <0.001).^12^ Garg et al. also reported that individual nutrition counselling and regular follow up can improve the nutritional status of pregnant women.^13^ Hence, the key finding of present study was consistent with the results of other studies that nutrition education improves the dietary knowledge, practices, and nutritional status of pregnant women.^10^-^13^

Bruins et al. reported that nutritional deficiency may persist even with primary nutrition interventions; therefore, secondary prevention can be more useful approach to address nutritional gaps. Furthermore, the authors stated that secondary prevention is potentially cost-effective mean to reduce healthcare cost and to improve the quality of life of individuals, but is often overlooked or underestimated.^14^ The results reported by Bookari et al. showed that Australian pregnant women valued the healthcare providers as the most reliable source of nutrition information; whereas media and social network were less reliable sources.^15^ The studies emphasized on the need of nutrition education by the healthcare workers; and on useful interaction between women and the healthcare providers.^10^,^15^ Similarly, the nutrition education intervention given by the clinical nutritionist in this study evidenced the usefulness of nutrition counselling and suggests that pregnant women seeking antenatal care should be provided with individual nutrition counselling.

### Limitations of study

It includes small sample size without control, short duration of study and follow up period, study population mainly from poor class and limited resources to provide prescribed supplements to the participants.

### Conclusion

Overall, nutrition counselling showed positive effects on nutritional status of pregnant women. Thus, the nutrition counseling must be an essential part of antenatal care for all pregnant women in the setting.

### Authors’ Contribution

**RK:** Concept and design, data collection, critical review, and final approval.

**MA:** Data collection and interpretation, manuscript writing, and final approval.

**MN:** Data transformation and interpretation, critical review, and final approval.

**TR:** Data entry, statistical analysis, critical review and final approval.
